# Surgical Versus Nonsurgical Management of Pancreatic Neuroendocrine Tumors: A Systematic Review and Meta-Analysis

**DOI:** 10.1245/s10434-025-17819-3

**Published:** 2025-07-24

**Authors:** Elias Khajeh, Mohammadamin Shahrbaf, Leonidas Apostolidis, Christoph W. Michalski, Martin Loos, Arianeb Mehrabi

**Affiliations:** 1 Department of General, Visceral and Transplantation Surgery, Heidelberg University, Heidelberg, Germany; 2 Department of Medical Oncology, National Center for Tumor Diseases (NCT) Heidelberg, Heidelberg University Hospital, Heidelberg, Germany

**Keywords:** Neuroendocrine tumors, Pancreatic neoplasms, Gastro-enteropancreatic tumor, Pancreatic neuroendocrine tumors, Surgical oncology

## Abstract

**Background:**

Pancreatic neuroendocrine tumors (PNETs) are a rare type of pancreatic cancer. Surgical resection is the primary curative treatment option, but whether this is the optimal treatment remains debatable. To address this question, we compared surgical versus nonsurgical management of PNETs.

**Patients and Methods:**

We searched the Medline (via PubMed), Scopus, Web of Science, and Embase databases through the end of 2023. Studies that compared surgical and nonsurgical interventions for PNETs across all grades, stages, and sizes and that reported survival outcomes were included. The primary endpoint was overall survival. The meta-analysis was performed using STATA software.

**Results:**

Seventy-seven articles (including 62 654 patients) were included in the final analysis. The pooled hazard ratio (HR) for mortality of surgical versus nonsurgical management was 0.30 (95% confidence interval [CI], 0.26–0.34; *P* < 0.001). Compared with nonsurgical management, surgical management was associated with better 1-year (94% vs. 78%), 3-year (85% vs. 57%), 5-year (77% vs. 46%), and 10–year (64% vs. 34%) survival. Pooled HRs for the mortality of patients who underwent surgery versus nonsurgical management for functional, nonfunctional, low grade (G1/G2), small (< 2 cm), and metastatic/locally advanced PNETs were 0.30, 0.30, 0.31, 0.26, and 0.33 respectively (*P* < 0.001). Surgical management of small PNETs (< 1 cm) showed comparable survival rates between surgical and nonsurgical approaches.

**Conclusions:**

Surgical PNET management had better outcomes than nonsurgical PNET management and should be considered the primary treatment option for resectable PNETs, However, nonsurgical management may be a reasonable option for select patients with small (<1 cm), asymptomatic PNETs.

**Supplementary Information:**

The online version contains supplementary material available at 10.1245/s10434-025-17819-3.

Pancreatic neuroendocrine tumors (PNETs) are rare malignancies that arise from hormone-producing cells in the pancreas.^1^ PNETs account for only 1–2% of all pancreatic neoplasms and are classified as functional or nonfunctional on the basis of their hormone-producing ability.^2^ Nonfunctional tumors are often asymptomatic and discovered incidentally during routine imaging, whereas functional tumors cause symptoms such as abdominal pain, diarrhea, flushing, and hypoglycemia.^3^ The incidence of PNETs has increased in recent years to an estimated 1.5 cases per 100,000 individuals annually,^4^ and the mortality rate is high (~58% 5-year survival) because of late diagnosis, metastasis, tumor characteristics, and treatment challenges,^5,6^

The poor prognosis of PNETs has spurred research into different treatment options, including surgery, chemotherapy, and radiation therapy.^7^ Surgical resection is the primary curative treatment option for PNETs; however, nonsurgical management, such as watchful waiting or systemic/interventional management, have also been considered for some patients.^8^ Despite the benefits of surgical management, there is an ongoing debate about the optimal PNET treatment approach. Some evidence suggests that nonsurgical management can be as effective as surgical management for small nonfunctioning PNETs as these tumors grow slowly.^9^ Other studies have shown desirable outcomes for nonsurgical management of well-differentiated, low-grade PNETs.^10,11^ Nonsurgical management has also shown efficacy in patients with PNET with end-stage disease.^12^

The European Society of Endocrine Surgeons recently recommended surgical management of advanced PNETs. However, no quantitative data were provided to support this evidence-based recommendation and only advanced PNETs were discussed.^13^ To address this gap, we conducted a systematic review and meta-analysis to compare survival outcomes following surgical and nonsurgical management of PNETs based on functionality, grade, tumor size, and distant metastasis.

## Patients and Methods

This systematic review and meta-analysis compared the survival outcomes of surgical versus nonsurgical management of PNETs. The study adhered to the Preferred Reporting Items for Systematic Reviews and Meta-Analyses (PRISMA) statement^14^ and the recommendations from the Study Center of the German Society of Surgery.^15^ The study protocol was registered with the International Prospective Register of Systematic Reviews (PROSPERO) under registration number CRD42023432119.

### Literature Search

A systematic search of Medline (via PubMed), Web of Science, Scopus, and Embase was performed for articles published until the end of 2023. Other relevant articles were also searched for in the reference lists of identified studies. No restrictions were placed on publication time. The following search terms were used:

((“surgical”[Title/Abstract] OR “surgery”[Title/Abstract] OR “operation”[Title/Abstract] OR “resection”[Title/Abstract] OR “enucleation”[Title/Abstract] OR “pancreatectomy”[Title/Abstract] OR “pancreatoduodenectomy”[Title/Abstract]) AND (“nonsurgical”[Title/Abstract] OR “nonsurgical”[Title/Abstract] OR “conservative”[Title/Abstract] OR “wait and watch”[Title/Abstract] OR “non surgery” [Title/Abstract] OR “chemotherapy”[Title/Abstract] OR “endoscopy”[Title/Abstract] OR “EUS”[Title/Abstract] OR “interventional radiology”[Title/Abstract] OR “drug therapy”[Title/Abstract] OR “radiotherapy”[Title/Abstract] OR “molecular targeted therapy”[Title/Abstract] OR “ablation”[Title/Abstract] OR “radiofrequency”[Title/Abstract] OR “embolization”[Title/Abstract] OR “chemoembolization”[Title/Abstract])) AND (“pancreas neuroendocrine tumor” [Title/Abstract] OR “neuroendocrine tumor”[Title/Abstract] OR “gastroenteropancreatic”[Title/Abstract] OR “PNET”[Title/Abstract] OR “P-NET”[Title/Abstract] OR “panNET”[Title/Abstract] OR “pan-NET”[Title/Abstract]).

The full search strategy is presented in the Supplementary Materials.

### Eligibility Criteria

The eligibility criteria for this study were formulated using the population, intervention, comparator, outcomes, and study design framework:^16^

- Population: patients with PNETs
- Intervention: surgical management
- Comparator: nonsurgical management
- Outcomes: hazard ratio (HR) for survival and the 1-, 3-, 5-, and 10-year survival rates following surgical management and nonsurgical management
- Study design: retrospective and prospective observational studies that reported survival data following surgical and nonsurgical management of PNETs

Studies that compared surgical and nonsurgical management of PNETs together with survival outcomes without any restrictions based on patient age, race, gender, or date were included. The exclusion criteria were studies on non-human populations, case reports, case series with fewer than ten patients, conference papers, systematic reviews/meta-analyses, and non-English papers.

### Study Selection and Data Extraction

The search results were reviewed by two researchers, (E.K. and M.S.), who first screened the titles and abstracts of the articles. In cases of disagreement, a third reviewer (A.M.) screened the article and decided whether it should be included. Duplicate and irrelevant articles were excluded. The full-text of included articles meeting the eligibility criteria were screened and the following data were extracted: author’s name, year of publication, country of the study, demographic characteristics of participants, and survival indices including the HR and the 1-, 3-, 5-, and 10-year survival rates following surgical and nonsurgical management. Numerical data were extracted from the Kaplan–Meier curve using GetData Graph Digitizer (version 2.24) software.

### Risk of Bias Assessment

The risk of bias was assessed using the risk of bias in non-randomized studies of interventions (ROBINS-I) tool.^17^ ROBINS-I calculates the overall risk of bias based on the following seven domains: confounding, selection of participants, classification of exposures, deviation from intended interventions, missing data, measurement of outcomes, and selection of the reported result. The risk of bias was defined as low, medium, or serious. The quality of evidence was assessed using the GRADE approach, which considers the risk of bias, inconsistency, indirectness, imprecision, and publication bias.^18^

### Statistical Analysis

The meta-analysis was conducted using STATA software (version 17). A random-effects model using the DerSimonian–Laird method was employed to calculate the pooled effect size and 95% confidence intervals (CIs). Heterogeneity among studies was assessed using the *Q* test and I_2_ index statistic. Sensitivity and subgroup analyses were also conducted on the basis of tumor activity, tumor grade, tumor size, distant metastasis, molecular subtypes, familial syndrome, different surgical and nonsurgical managements, tumor location, and cause-specific mortality. When the evidence was not enough for conducting a meta-analysis, qualitative analysis was performed. Publication bias was assessed using the Egger linear regression test, Begg rank correlation test, and funnel plots.

## Results

**Fig. 1:**
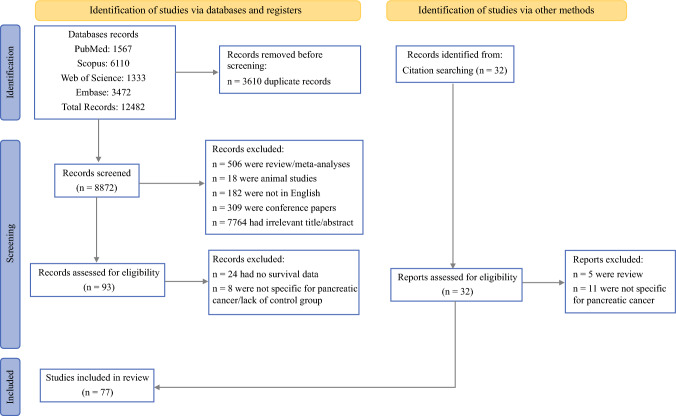
PRISMA flowchart of the study

We retrieved 12,482 articles. After removing 3610 duplicates, 8872 articles were screened. Of those, 506 were reviews/meta-analyses, 18 were animal studies, 182 were non-English studies, 309 were conference papers, and 7764 had irrelevant titles/abstracts. Overall, 93 articles were eligible for full-text screening, of which 24 articles were excluded for lacking survival data following surgical and nonsurgical PNET management and 8 articles were excluded for lacking specific data on the PNETs. After exclusion, 77 articles were included in the final analysis.^5,19–94^ The PRISMA study flowchart is presented in Fig. 1. The analysis included 62,654 patients with PNETs, 51.5% of whom were males. The studies included in the analysis were published between 2001 and 2023, and the retrospective data spanned from 1956 to 2022. Sample sizes ranged from 27 to 6088 patients; 53.2% of these underwent surgery and 82.6% had nonfunctional PNETs. In total, 37 studies reported follow-up periods with a median of 43 months (range, 12–127 months). The study characteristics are presented in Table 1. The risk of bias analysis is presented in Supplementary Materials (Table S1). All studies except three had a serious risk of bias, which was mainly due to the retrospective nature of studies and confounding variable bias.

**Table 1:** General characteristics of included studies

No.	Study	Year	Country	Duration	Sample size	M/F	F/NF	S/nS	nS management	FU (months)
1	Solorzano et al. ^18^	2001	USA	1988–1999	163	91/72	0/163	62/101	Systematic chemotherapy, chemoembolization	NM
2	Chu et al. ^19^	2002	USA	1970–2001	50	28/22	21/29	31/19	Systematic chemotherapy, chemoembolization, observation	35
3	Tomassetti et al. ^20^	2005	Italy	1978–2003	83	37/46	20/63	40/43	Systematic chemotherapy, chemoembolization, SSA	30
4	Kouvaraki et al. ^21^	2006	USA	NM	55	28/27	35/20	38/17	Observation	52
5	Triponez et al. ^22^	2006	France	1956–2003	108	38/70	0/108	43/65	Binary outcome (yes/no)	52
6	Nguyen et al. ^23^	2007	USA	1989–1999	73	37/36	22/51	42/31	Systematic chemotherapy, chemoembolization, SSA, radiation	41
7	Shurr et al. ^24^	2007	Germany	1987–2004	62	32/30	16/46	45/17	Systematic chemotherapy, SSA	30
8	Fischer et al. ^25^	2008	Germany	1994–2006	118	66/52	11/107	102/16	Systematic chemotherapy	20
9	Ruiz–Tovar et al. ^26^	2008	Spain	1985–2007	49	22/27	36/13	43/6	Binary outcome (yes/no)	80
10	Bettini et al. ^27^	2009	Italy	1990–2004	51	24/27	0/51	19/32	Binary outcome (yes/no)	26
11	Hill et al. ^28^	2009	USA	1988–2002	727	393/334	114/613	310/417	Binary outcome (yes/no)	26
12	Franko et al. ^29^	2010	USA	1973–2004	1590	889/701	0/1590	735/855	Binary outcome (yes/no)	NM
13	Ito et al. ^30^	2010	USA	1992–2006	73	36/37	26/47	50/23	Binary outcome (yes/no)	43
14	Wang et al. ^31^	2011	China	1995–2010	27	16/11	4/23	18/9	Systematic chemotherapy	40
15	Martin–Perez et al. ^32^	2013	Spain	2001–2010	483	244/239	171/312	318/165	Systematic chemotherapy, chemoembolization, SSA, PRRT	NM
16	Zerbi et al. ^33^	2013	Italy	2004–2007	140	77/63	16/124	100/40	Systematic chemotherapy, SSA, PRRT, ablation	21
17	Bertani et al. ^34^	2014	Italy	1998–2008	43	20/23	11/32	12/31	Systematic chemotherapy, SSA, PRRT	60
18	Crippa et al. ^35^	2014	Italy	1990–2009	355	182/173	0/355	121/61	Observation	44
19	Gratian et al. ^36^	2014	USA	1998–2011	1367	606/761	0/1367	999/368	Binary outcome (yes/no)	62
20	Dumont et al. ^37^	2015	France	1984–2012	42	22/20	3/39	28/14	Systematic chemotherapy	72
21	Hüttner et al. ^38^	2015	Germany	2004–2011	442	247/195	0/442	75/367	Binary outcome (yes/no)	24
22	Partelli et al. ^39^	2015	Italy	2000–2011	166	92/74	14/152	91/75	Observation	41
23	Sharpe et al. ^40^	2015	USA	1998–2006	380	162/218	0/380	309/71	Systematic chemotherapy, radiation	33
24	van Vliet et al. ^41^	2015	The Netherlands	2000–2011	29	NM	0/29	9/20	PRRT	NM
25	Bertani et al. ^42^	2016	Italy	1996–2013	94	47/47	27/67	31/63	Systematic chemotherapy, SSA, PRRT	51
26	Haugvik et al. ^43^	2016	Norway	1998–2012	90	55/35	NM	12/78	Systematic chemotherapy	12
27	Keutgen et al. a ^44^	2016	USA	1973–2011	882	488/394	0/882	303/579	Binary outcome (yes/no)	NM
28	Keutgen et al. b ^45^	2016	USA	1973–2011	401	195/206	401/0	273/128	Binary outcome (yes/no)	NM
29	Massironi et al. ^46^	2016	Italy	2007–2014	36	14/22	11/25	21/15	Observation, SSA, ablation	42
30	Partelli et al. ^47^	2016	Italy	1997–2013	60	30/30	0/60	27/33	Observation	126
31	Rosenberg et al. ^48^	2016	USA	1999–2014	35	18/17	0/35	20/15	Observation	28
32	Sadot et al. ^49^	2016	USA	1993–2013	181	95/86	0/181	77/104	Observation	50
33	Zhang et al. ^50^	2016	USA	1998–2014	249	130/119	0/249	193/56	Observation	57
34	Bertani et al. ^51^	2017	Italy	1994–2013	93	47/46	71/22	63/30	Systematic chemotherapy, SSA, PRRT	88
35	Citterio et al. ^52^	2017	Italy	1979–2005	36	17/19	36/0	24/12	Systematic chemotherapy, SSA	127
36	Prakash et al. ^53^	2017	USA	2000–2012	29	21/8	NM	14/15	Systematic chemotherapy	88
37	Tao et al. ^54^	2017	China	2010–2014	191	118/73	0/191	47/144	Binary outcome (yes/no)	NM
38	Chawla et al. ^55^	2018	USA	1998–2012	4038	2254/1784	NM	351/3687	Binary outcome (yes/no)	NM
39	Genc et al. ^56^	2018	Netherlands	2008–2013	405	195/210	NM	255/150	Systematic chemotherapy, SSA, PRRT	26
40	Nell et al. ^57^	2018	Netherlands	1990–2014	152	68/84	0/152	53/99	Observation	71
41	Li et al. ^58^	2019	China	2004–2014	653	325/328	0/653	189/459	Binary outcome (yes/no)	NM
42	Tierney et al. ^59^	2019	USA	2004–2014	6088	NM	NM	460/5628	Binary outcome (yes/no)	NM
43	Wu et al. ^60^	2019	China	2000–2014	4766	2574/2192	0/476	2436/2330	Binary outcome (yes/no)	NM
44	Ye et al. ^61^	2019	China	2004–2015	1974	1116/858	0/1974	392/1582	Binary outcome (yes/no)	19.5
45	Zhang et al. ^62^	2019	China	1973–2015	210	117/93	NM	62/148	Systematic chemotherapy, SSA, PRRT	NM
46	Zheng et al. ^63^	2019	China	2010–2015	501	NM	NM	168/333	SSA, PRRT	NM
47	Assi et al. ^64^	2020	USA	2004–2015	2004	1022/982	0/2004	1781/223	Binary outcome (yes/no)	26
48	Chivukula et al. ^65^	2020	USA	2004–2014	3243	1553/1690	84/3159	2552/691	Binary outcome (yes/no)	34
49	Fathi et al. ^66^	2020	USA	1988–2012	1787	1027/760	0/1787	968/819	Binary outcome (yes/no)	NM
50	Fujimori et al. ^67^	2020	Japan	1987–2018	245	115/130	66/179	188/57	Systematic chemotherapy, SSA, PRRT	NM
51	Kurita et al. ^68^	2020	Japan	1998–2018	75	34/41	0/75	52/23	Observation	66
52	Powers et al. ^69^	2020	USA	2007–2015	709	336/373	0/709	628/81	Binary outcome (yes/no)	24
53	Sada et al. ^70^	2020	USA	1973–2015	121	52/69	121/0	88/33	Binary outcome (yes/no)	NM
54	Kaemmerer et al. ^71^	2021	Germany	2004–2014	335	NM	NM	148/187	PRRT	NM
55	Shaib et al. ^72^	2021	USA	2004–2014	620	NM	NM	111/509	Systematic chemotherapy, radiation	NM
56	Sun et al. ^73^	2021	China	2004–2015	2637	1403/1234	0/2637	2147/490	Binary outcome (yes/no)	NM
57	Tsilimigars et al. ^74^	2021	USA	2004–2015	969	523/446	0/969	48/921	Chemoembolization, ablation	NM
58	Bingmer et al. ^75^	2022	USA	2004–2016	3459	1971/1488	0/3459	2743/716	Binary outcome (yes/no)	45
59	Cai et al. ^76^	2022	China	2004–2017	990	NM	130/660	667/323	Observation	NM
60	Guo et al. ^77^	2022	China	2010–2015	637	308/329	0/637	564/73	Observation	56
61	Kaur et al. ^78^	2022	USA	1975–2016	347	159/188	347/0	193/154	Systematic chemotherapy	NM
62	Kjaer et al. ^79^	2022	USA	1985–2019	194	118/76	43/151	65/129	Systematic chemotherapy, SSA, PRRT	52
63	Krogh et al. ^80^	2022	Denmark	2011–2021	174	91/83	26/148	91/83	Observation, SSA	48
64	Liu et al. ^81^	2022	China	1975–2018	3453	1906/1547	340/3113	2857/596	Binary outcome (yes/no)	NM
65	Minczeles et al. ^82^	2022	Netherlands	2000–2019	49	25/24	3/43	26/23	PRRT	66
66	Mou et al. ^83^	2022	China	2000–2017	536	323/213	5/531	214/322	Binary outcome (yes/no)	43
67	So et al. ^84^	2022	Korea	2000–2018	285	156/129	0/285	188/97	Ablation (EUS–EA)	NM
68	Wu et al. ^85^	2022	China	2010–2015	751	346/405	0/751	670/81	Systematic chemotherapy, radiation	NM
69	Yang et al. a ^86^	2022	China	2000–2018	1621	783/838	0/1621	1350/271	Binary outcome (yes/no)	NM
70	Yang et al. b ^87^	2022	China	1998–2018	2387	1101/1286	1181/1206	1834/553	Systematic chemotherapy, radiation	NM
71	Ye et al. ^88^	2022	China	2004–2015	1006	465/541	0/1006	151/855	Binary outcome (yes/no)	NM
72	Zhu et al. ^89^	2022	China	1973–2015	681	332/349	0/681	559/122	Binary outcome (yes/no)	NM
73	Amini et al. ^90^	2023	USA	2010–2019	2127	1193/934	0/2127	707/1420	Systematic chemotherapy	NM
74	Crinò et al. ^91^	2023	Multi–Center	2014–2022	304	109/195	304/0	193/111	Ablation (EUS–RFA)	30
75	Heng et al. ^92^	2023	China	2004–2016	1824	969/855	0/1824	1408/416	Systematic chemotherapy, radiation	NM
76	Lin et al. ^93^	2023	China	2004–2015	1102	550/552	0/1102	965/137	Binary outcome (yes/no)	44
77	Luo et al. ^94^	2023	China	2000–2017	142	74/68	142/0	100/42	Binary outcome (yes/no)	NM

M, male; F, female; F, functional tumor; NF, nonfunctional tumor; S, surgery; nS, non-surgery; FU, follow–up; SSA, somatostatin analog; PRRT, peptide receptor radionuclide therapy; EUS–EA, endoscopic ultrasound guided ethanol ablation; EUS–RFA, endoscopic ultrasound guided radiofrequency ablation

### Overall Analysis

**Fig. 2:**
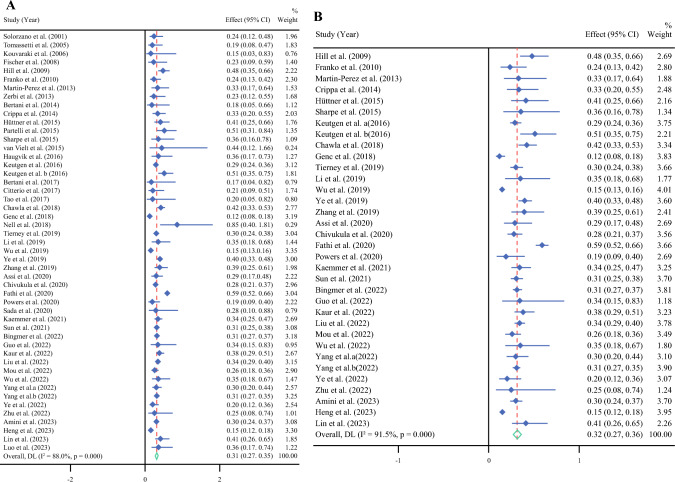
Mortality hazard ratio for survival of surgical managements in PNETs in comparison with nonsurgical management: **A** overall analysis and **B** after removing studies with sample size < 200 or follow-up < 24 months

A meta-analysis of 49 studies comprising 55,960 patients (53.5% of whom underwent surgery) with a median follow-up of 34 months (range, 12–127 months) was conducted to determine the pooled mortality HRs following surgical and nonsurgical management of PNETs. The pooled HR was 0.308 (95% CI, 0.268–0.347; *P* < 0.001; *I*^2^ = 88%), indicating that surgical resection significantly improves survival outcomes for patients with PNETs compared with nonsurgical management (Fig. 2a). In the sensitivity analysis, studies with fewer than 200 patients and those with the median follow-up < 24 months were removed. In this analysis, the pooled mortality HR of 34 remaining studies was 0.316 (95% CI, 0.272–0.360; *P* < 0.001; *I*^2^ = 91.5%). The sensitivity analyses confirmed that the overall conclusions were robust, with no significant changes observed (Fig. 2b).

A pooled analysis of 69 studies was conducted to assess the 1-, 3-, and 5-year survival rate in patients with PNETs. The analysis included 52,844 patients, 51% of whom underwent surgery. The 1-year survival rate of patients with PNET was 94.2% (95% CI, 92.1–96.2%; *I*^2^ = 100%) following surgery and 77.6% (95% CI, 73.6–81.7%; *I*^2^ = 1005) following nonsurgical management. The 3-year survival rate of patients was 84.7% (95% CI, 81–88.5%; *I*^2^ = 100%) following surgical management and 56.9% (95% CI, 50.5–63.2%; *I*^2^ = 100%) following nonsurgical management. The 5-year survival rate was 76.7% (95% CI, 72.5–81%; *I*^2^ = 100%) following surgical management and 46.1% (95% CI; 39.4–52.7%; *I*^2^ = 100%) following nonsurgical management.

A pooled analysis of 41 studies comprising 33,331 patients (56.4% of whom underwent surgery), was conducted to assess the 10-year survival rate in patients with PNETs. The 10-year survival was 64.3% (95% CI, 56.8–71.8%; *I*^2^ = 100%) following surgical management and 33.8% (95% CI, 24.4–43.1%; *I*^2^ = 100%) following nonsurgical management.

### Subgroup Analyses

#### Functional PNETs

##### All Functional PNETs

**Fig. 3:**
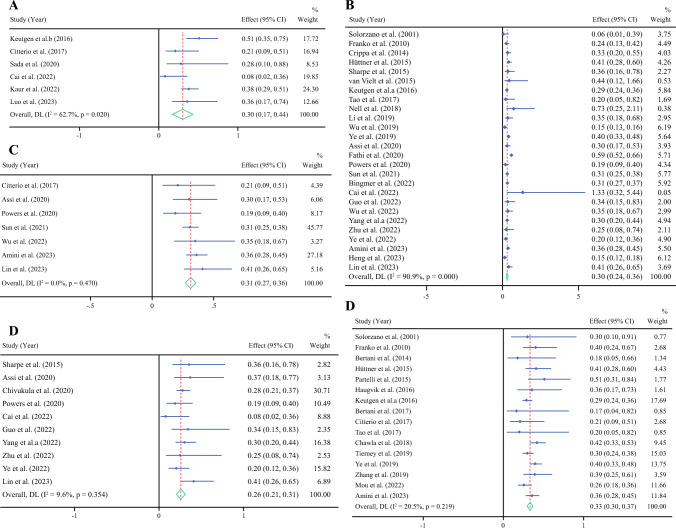
Mortality hazard ratio for surgical management in PNETs: **A** functional PNETs, **B** nonfunctional PNETs, **C** G1/G2 PNETs, **D** PNETs < 2 cm, and **E** metastatic//locally advanced PNETs

Included studies reported outcomes of all functional PNETs in a single group without subgroup analysis based on specific functional PNET types, such as insulinoma, VIPoma, glucagonoma, and gastrinoma. The pooled analysis of six studies including 1177 patients (57.6% of whom underwent surgery) over a median follow-up of 127 months was conducted to determine the pooled mortality HR following surgical resection versus nonsurgical management of functional PNETs. The HR was 0.304 (95% CI, 0.169–0.439; *P* < 0.001; *I*^2^ = 62.7%; Fig. 3a). After analyzing four studies involving 883 patients (66.8% of whom underwent surgery), the 1-year survival rate was 96.3% (95% CI, 92.8–99.8%; *I*^2^ = 100%) following surgical management and 82.6% (95% CI, 70.8–94.5%; *I*^2^ = 100%) following nonsurgical management. Surgical management was associated with a 3-year survival rate of 92.3% (95% CI, 87.6–97.1%; *I*^2^ = 100%) whereas nonsurgical management was associated with a 3-year survival rate of 59% (95% CI, 55.9–62%; *I*^2^ = 100%). The 5-year survival rate was 87.6% (95% CI, 79.8–95.5%; *I*^2^ = 100%) for surgical management and 50.6% (95% CI, 27.2–73.9%; *I*^2^ = 100%) for nonsurgical management. The 10-year survival rate was 70.8% (95% CI, 59.8–81.7%; *I*^2^ = 100%) following surgical management and 32.8% (95% CI, 25.3–40.3%; *I*^2^ = 100%) following nonsurgical management.

##### Functional PNETs < 2 cm

A single study reported the HR for mortality following surgical resection versus nonsurgical management for functional PNETs < 2 cm. The HR for surgery was 0.080 (95% CI, 0.020–0.360). The 1-year survival rate was 89.4% for the surgical group and 70% for the nonsurgical group. At 3 years, the survival rates were 75.3% for the surgical group and 50% for the nonsurgical group. The 5-year survival rates were 62.4% for the surgical group and 20% for the nonsurgical group. Also, the 10-year survival rate was 22.3% following surgical management and 0% following nonsurgical management.

#### Nonfunctional PNETs

##### All Nonfunctional PNETs

The mortality HRs of patients with nonfunctional PNETs were compared following surgical and nonsurgical management in a pooled analysis of 26 studies comprising 33,479 patients, 60.6% of whom underwent surgery. The HR was 0.302 (95% CI, 0.243–0.360; *P* < 0.001; *I*^*2*^ = 90.9%; Fig. 3b). After analyzing 31 studies comprising 28,969 patients (60.3% of whom underwent surgery), the 1-year survival was 94.6% (95% CI, 92.3–96.9%; *I*^2^ = 100%) following surgical management and 81.8% (95% CI, 78.9–84.8%; *I*^2^ = 100%) following nonsurgical management. The 3-year survival rate was 86.2% (95% CI, 82.3–90.2%; *I*^2^ = 100%) following surgical management and 63.8% (95% CI, 55.5–72.2%; *I*^2^ = 100%) following nonsurgical management. The 5-year survival rate was 77.1% (95% CI, 71.1–83.1%; *I*^2^ = 100%) following surgical management and 53.9% (95% CI, 44.4–63.4%; *I*^2^ = 100%) following nonsurgical management. Finally, the 10-year survival rate was 65.3% (95% CI, 54.1–76.6%; *I*^2^ = 100%) following surgical management and 46.6% (95% CI, 29.9–63.4%; *I*^2^ = 100%) following nonsurgical management.

##### Nonfunctional PNETs < 2 cm

The mortality HRs of patients with small nonfunctional PNETs were compared following surgical and nonsurgical management in a pooled analysis of ten studies comprising 12,564 patients, 75% of whom underwent surgery. The HR was 0.330 (95% CI, 0.220–0.440; *P* < 0.001; *I*^2^ = 84%; Fig. S1). After analyzing 15 studies comprising 14,532 patients (75% of whom underwent surgery), the 1-year survival was 96.2% (95% CI, 95.4–96.9%; *I*^2^ = 100%) following surgical management and 87.3% (95% CI, 85.7–88.8%; *I*^2^ = 100%) following nonsurgical management. The 3-year survival rate was 90% (95% CI, 86.2–93.8%; *I*^2^ = 100%) following surgical management and 73.3% (95% CI, 61.3–85.3%; *I*^2^ = 100%) following nonsurgical management. The 5-year survival rate was 84.8% (95% CI, 80.2–89.5%; *I*^2^ = 100%) following surgical management and 65.1% (95% CI, 56.8–73.5%; *I*^2^ = 100%) following nonsurgical management. Finally, the 10-year survival rate was 69.5% (95% CI, 57.1–81.8%; *I*^2^ = 100%) following surgical management and 58.6% (95% CI, 33.2–83.9%; *I*^2^ = 100%) following nonsurgical management.

#### Low Grade and Intermediate Grade PNETs (G1 and G2 tumors)

A pooled analysis of seven studies comprising 7666 patients (83.9% of whom underwent surgery), was conducted to determine mortality HR of surgical versus nonsurgical management for G1 and G2 PNETs over a median follow-up of 36 months. The HR was 0.314 (95% CI, 0.270–0.358; *P* < 0.001; *I*^2^ = 0%; Fig. 3c). After analyzing 14 studies with a total of 10,946 patients (68% of whom underwent surgery), the 1-year survival rate was 95.8% (95% CI, 93.9–97.7%; *I*^2^ = 100%) for surgical management and 85.1% (95% CI, 77.7–92.5%; *I*^2^ = 100%) for nonsurgical management. Surgical management was associated with a 3-year survival of 89.9% (95% CI, 83.9–96%; *I*^2^ = 100%) while nonsurgical management was associated with a 3-year survival of 64.6% (95% CI, 54.2–75%; *I*^2^ = 100%). Surgical management was associated with a 5-year survival of 83% (95% CI, 75–91.1%; *I*^2^ = 100%) while nonsurgical management was associated with a 5-year survival rate of 54.2% (95% CI, 41.1–67.2%; *I*^2^ = 100%). Of 4550 patients (81.9% of whom underwent surgery), surgical management was associated with a 79.3% 10-year survival rate (95% CI, 69.9–88.7%; *I*^2^ = 100%) while nonsurgical management was associated with a 10-year survival rate of 39.8% (95% CI, 5–74.5%; *I*^2^ = 100%). A subgroup analysis of patients with G1 PNET also showed improved survival outcomes in patients undergoing surgery compared with nonsurgical management (Fig. S2).

#### Tumor Size

##### < 2 cm PNETs

A pooled analysis of ten studies that included 12,373 patients (77% of whom underwent surgery) was conducted to determine the mortality HR following surgical versus nonsurgical management of small PNETs (< 2 cm). The HR was 0.259 (95% CI, 0.206–0.312; *P* < 0.001; *I*^2^ = 9.6%; Fig. 3d). After analyzing 19 studies with a total of 16,958 patients (70% of whom underwent surgery), surgical management was associated with a 1-year survival rate of 96% (95% CI, 95.4–96.7%; *I*^2^ = 100%) whereas nonsurgical management was associated with a 1-year survival rate of 85.2% (95% CI, 77.9–92.4%; *I*^2^ = 100%). Surgical management was associated with a 3-year survival rate of 90% (95% CI, 87–93%; *I*^2^ = 100%) whereas nonsurgical management was associated with a 3-year survival rate of 71.1% (95% CI, 58.6–83.7%; *I*^2^ = 100%). Surgical management was associated with a 5-year survival rate of 86% (95% CI, 82.3–89.8%; *I*^2^ = 100%) and nonsurgical management was associated with a 5-year survival rate of 62.9% (95% CI, 50.1–75.6%; *I*^2^ = 100%). With regard to 10-year survival, surgical management was associated with a 10-year survival rate of 72.5% (95% CI, 62.9–82.2%; *I*^2^ = 100%), whereas nonsurgical management was associated with a 10-year survival rate of 60.5% (95% CI, 36.2–84.8%; *I*^2^ = 100%).

##### < 1 cm PNETs

A pooled analysis of three studies that included 2417 patients (79.5% of whom underwent surgery) was conducted to determine the mortality HR following surgical versus nonsurgical management of small PNETs (< 1 cm). The HR was 0.437 (95% CI, 0.089–0.785; *P* = 0.014; *I*^2^ = 59.7%; Fig. S3). Surgical management was associated with a 1-year survival rate of 97% (95% CI, 96–97%) whereas nonsurgical management was associated with a 1-year survival rate of 97% (95% CI, 91–100%). Surgical management was associated with a 3-year survival rate of 91% (95% CI, 88–94%), whereas nonsurgical management was associated with a 3-year survival rate of 89% (95% CI, 78–96%). Surgical management was associated with a 5-year survival rate of 86% (95% CI, 83–89%) and nonsurgical management was associated with a 5-year survival rate of 88% (95% CI, 76–96%). With regard to 10-year survival, surgical management was associated with a 10-year survival rate of 70% (95% CI, 68–72%), whereas nonsurgical management was associated with a 10-year survival rate of 69% (95% CI, 65–73%).

#### Metastatic/Locally Advanced PNETs

In the assessment of metastatic and locally advanced PNETs, all patients who had locally advanced PNETs also had distant metastasis, and these two groups were simultaneously analyzed. A pooled analysis of 16 studies that included 17,505 patients with metastatic/locally advanced PNETs, 11.7% of whom underwent surgery, was conducted to determine the mortality HR of surgical versus nonsurgical management. After a median follow-up of 42 months, the HR was 0.335 (95% CI, 0.299–0.371; *P* < 0.001; *I*^2^ = 20.5%; Fig. 3e). After analyzing 26 studies with a total of 20,583 patients (15.3% surgery), surgical management was associated with a 1-year survival rate of 89% (95% CI, 84.6–93.5%; *I*^2^ = 100%), whereas nonsurgical management was associated with a 1-year survival rate of 68.2% (95% CI, 59.6–76.7%; *I*^2^ = 100%). Surgical management was associated with a 3-year survival rate of 73.4% (95% CI, 62–83.6%; *I*^2^ = 100%) and nonsurgical management was associated with a 3-year survival rate of 39.3% (95% CI, 30.6–48.1%; *I*^2^ = 100%). Surgical management was associated with a 5-year survival rate of 60.5% (95% CI, 53.3–67.7%; *I*^2^ = 100%), whereas nonsurgical management was associated with a 5-year survival rate of 27.2% (95% CI, 20.5–33.8%; *I*^2^ = 100%).

In an assessment of 10,168 patients (11.9% of whom underwent surgery), surgical management was associated with a 10-year survival rate of 37.3% (95% CI, 22.1–52.6%; *I*^2^ = 100%) and nonsurgical management was associated with a 10-year survival rate of 14.2% (95% CI, 9.2–19.3%; *I*^2^ = 100%).

### Qualitative Analysis

#### Molecular Subtypes

Subgroup analysis of surgical and nonsurgical management according to molecular biology differences between PNETs remains extremely limited in the current literature. Kouvaraki et al. identified MEN1 exon 2 mutations as being associated with higher progression rates.^22^ Several other studies, including Tomassetti et al.^21^ Schurr et al.^25^, Bertani et al.^35^, and Fujimori et al.^68^, reported Ki-67 indices, and Bertani et al. and Fujimori et al. found associations with worse prognosis, but none conducted molecular subgroup analyses comparing surgical and nonsurgical managements. Studies such as Fischer et al.^26^, Ito et al.^31^, and Wang et al.^32^ applied WHO histologic classifications, but again did not assess differential outcomes based on treatment modality. Other molecular mutations such as ATRX, DAXX, and telomere length alterations, were also not compared between surgical and nonsurgical management groups in the reviewed studies.

#### Familial Syndromes

Subgroup analysis of surgical versus nonsurgical managements in familial PNETs is limited and inconsistently reported across the literature. Only four studies focused exclusively on patients with multiple endocrine neoplasia type 1 (MEN1).^22,23,48,58^ These studies yielded mixed results. Triponez et al.^23^, Partelli et al.^48^, and Nell et al.^58^ found no survival benefit for surgery over observation in small, nonfunctioning PNETs in patients with MEN1. Meanwhile, Kouvaraki et al.^22^ reported improved survival with surgery. In addition, at least five studies^21,27,47,51,68^ mentioned the inclusion of familial syndromes such as MEN1 or von Hippel–Lindau (VHL) but did not provide dedicated subgroup analyses comparing treatment outcomes. The majority of studies either did not mention familial syndromes at all or only briefly noted single MEN1/VHL cases without further analysis, limiting the generalizability of their findings to hereditary cases.

#### Different Surgical Managements

Most studies evaluating surgery in PNETs did not perform detailed subgroup analyses comparing outcomes between different surgical techniques with nonsurgical managements. In the majority of publications, surgery was reported as a binary variable (yes/no), without specifying approach. Among studies that did list surgical types, few provided comparative outcome analyses. Wu et al.^85^ found that partial pancreatectomy had the best cancer-specific survival, with 5-year mortality of 1.87%, compared with 12.35% for total pancreatectomy, suggesting more conservative resections may be preferable when feasible. Similarly, Powers et al.^70^ reported the lowest mortality with enucleation. The choice of surgical procedure was generally influenced by tumor location, size, functionality, proximity to ducts/vessels, and patient comorbidities. There is no consensus on the optimal surgical approach, and most evidence lacks comparative effectiveness data.

#### Different Nonsurgical Managements

Most studies evaluating nonsurgical management in PNETs did not perform detailed subgroup analyses to compare the outcomes of various nonsurgical modalities. In the majority of studies, nonsurgical managements were reported as a binary category, without specifying or analyzing different therapeutic strategies such as somatostatin analogs (SSA), peptide receptor radionuclide therapy (PRRT), chemotherapy, or observation. Among the few studies that provided stratified data, PRRT ^42,57,72,82^ appeared to be associated with better survival outcomes compared with chemotherapy or observation in advanced/metastatic cases. SSA were commonly used, especially in small, low-grade tumors, but showed limited progression benefit unless combined with other agents.^53^ Observation alone was deemed safe in select patients with ≤ 2 cm, nonfunctioning, low-grade tumors, as demonstrated by Partelli et al.^48^, Kurita et al.^69^, Sadot et al.^50^, and Krogh et al.^81^, with minimal disease progression over long-term follow-up. In contrast, chemotherapy alone was generally associated with poorer outcomes, particularly in metastatic settings.^44,73,79^ In addition, interventional strategies such as endoscopic ultrasound (EUS)-guided ethanol ablation^84^ or radiofrequency ablation (RFA)^91^ were reported with good local control and minimal morbidity in select small, functional PNETs. Overall, nonsurgical therapy choice appears to depend on tumor functionality, grade, metastatic burden, and patient comorbidities, but the lack of head-to-head comparisons limits evidence-based selection of the best modality.

#### Tumor Location

Most studies on PNETs did not provide a detailed subgroup analysis comparing surgical versus nonsurgical management based on tumor location. When reported, tumors in the pancreatic head were generally associated with higher surgical risk and complication rates, particularly due to the complexity of pancreatoduodenectomy, whereas tumors in the body and tail were more frequently resected with lower morbidity, such as with distal pancreatectomy or enucleation.^38,41,83,84^ Keutgen et al.^45^, Li et al.^59^, Bingmer et al.^76^, and Heng et al.^92^ showed that body/tail location was independently associated with better overall survival. However, no consistent survival benefit of surgery versus nonsurgery stratified by location was demonstrated across most studies. Only Citterio et al.^53^, Wu et al.^61^, and Shaib et al.^73^ presented analyses supporting better survival with surgery in PNETs, particularly for tail tumors. In contrast, tumors in the pancreatic head were more frequently observed rather than resected owing to surgical complexity, and showed poorer outcomes when treated conservatively.^65,84^

#### Cause-Specific Mortality

Subgroup analyses comparing cause-specific mortality between surgical and nonsurgical management of PNETs remain limited. While many studies reported overall or cancer-specific survival, only a minority explicitly detailed the causes of death. Among those that did, tumor progression and liver metastasis were the most frequent causes of death in both surgical and nonsurgical groups.^19,22,33,47,81^ In Triponez et al.^23^, ten deaths were attributed to nonfunctioning pNET, while others were due to unrelated comorbidities. Chawla et al.^56^ highlighted significantly lower 90-d mortality in surgery versus metastasectomy-only groups, indicating surgery had lower early treatment-related deaths. In Tao et al.^55^, cancer-specific deaths were significantly reduced in the surgical group, and in Wu et al.^85^, nonsurgical patients had markedly higher 5-year cancer-specific mortality (49.2%) compared with surgical subgroups (as low as 1.87%). Sadot et al.^50^ and Zhang et al.^51^ found no confirmed PNET-related deaths in either group. Nell et al.^58^ reported more PNET-related deaths in the surgery group (three versus one), but most deaths in both groups were unrelated to cancer. While surgery is generally associated with improved overall and cancer-specific survival, direct comparisons of cause-of-death patterns between surgical and nonsurgical groups are largely unavailable, limiting definitive conclusions on the nature of mortality in each group.

### Survival Distribution and Survival Trend

**Fig. 4:**
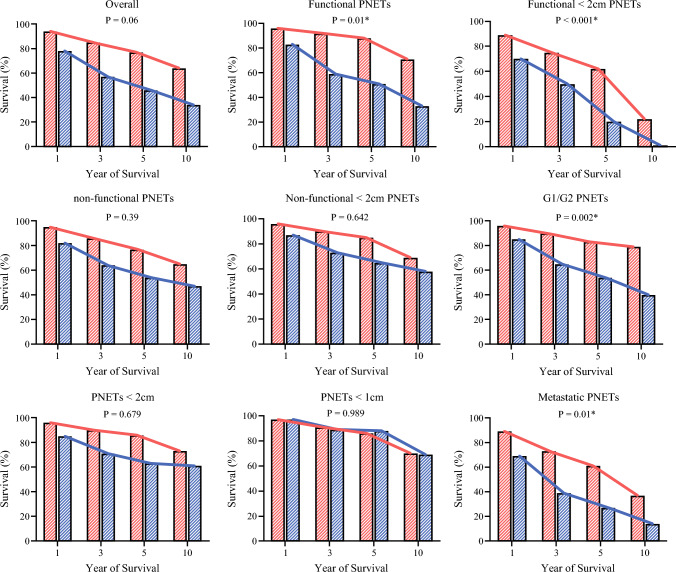
1-, 3-, 5-, and 10-year survival rate for different subgroups: survival differences in all time-points were significantly different (* *P* < 0.001); the trend line and *P*-value of the trend for survival in different subgroups are provided above

The distribution and trend of survival rates over time are presented in Fig. 4. Except for < 1 cm PNETs, the results showed that surgical management is superior to nonsurgical management in all subgroups and survival periods (*P* < 0.001). The trend analysis indicated that survival trends were significantly better after surgical management than after nonsurgical management of functional PNETs (*P* = 0.01), functional PNETs < 2 cm (*P* < 0.001), G1/G2 PNETs (*P* = 0.002), and metastatic PNETs (*P* = 0.01).

### Publication Bias Analysis

**Fig. 5:**
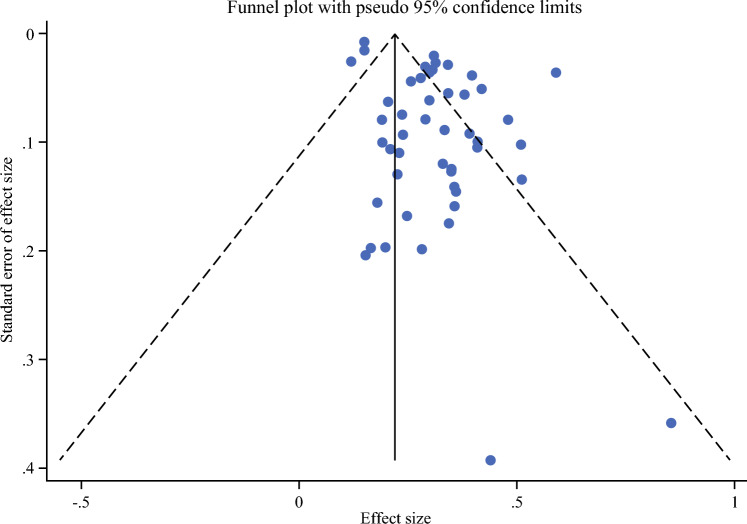
Funnel plot for publication bias analysis based on the primary endpoint

Publication bias for the main outcome was assessed using Egger’s and Begg’s tests. Egger’s test yielded a significant result (coefficient = 2.05, standard error [SE] = 0.57, *P* < 0.001), and Begg’s test indicated significant asymmetry as well (Kendall’s score = −352, standard deviation [SD] = 115.99, *P* = 0.002). These results provide statistical evidence of publication bias in the included studies. Figure 5 presents a funnel plot that further supports these results. As it can be seen, the plot shows asymmetry, suggesting that studies with nonsignificant or negative findings may be underreported or unpublished. In addition, the asymmetrical spread and vertical skew imply differential ranking or selective inclusion of studies, likely favoring those with larger effect sizes or statistically significant outcomes.

### Grading of Evidence

The quality assessment based on the GRADE approach is presented in the Supplementary Materials (Table S2). The quality of evidence was mainly low and very low because of high heterogeneity, indirectness, and high risk of bias.

## Discussion

In this study, we compared survival outcomes following surgical versus nonsurgical management of PNETs. We found that surgical management is associated with better survival outcomes than nonsurgical management, even in cases of small, metastatic/locally advanced, and low-grade PNETs. To the best of our knowledge, this is the largest meta-analysis on this topic to date, including over 48,000 PNET cases from the general population. These findings will significantly impact future PNET treatment strategies.

There are several surgical techniques for managing PNETs. These techniques vary depending on the size, location, and stage of the tumor, and include enucleation, pancreaticoduodenectomy, distal pancreatectomy, and total pancreatectomy. These procedures have been performed using laparoscopic-assisted and robotic-assisted techniques.^95,96^ Surgical resection has been associated with postoperative morbidity and mortality and the risk of long-term exocrine and endocrine insufficiency associated with pancreatic resection.^97^ A systematic review showed that observation rather than surgery is an acceptable management for small and asymptomatic PNETs.^98^

Nonsurgical management includes watchful waiting, chemotherapy, radiation therapy, molecular targeted therapy, and ablation techniques^99^, and has gained much attention in recent years. Watchful waiting has been shown to be a safe management for small (< 2 cm) and sporadic PNETs and only a small proportion of these patients showed tumor progression.^100,101^ The immune checkpoint inhibitor pembrolizumab and oral multitargeted tyrosine kinase inhibitor sunitinib have both been approved for the treatment of PNETs.^12^ Moreover, ablation techniques such as radiofrequency ablation and microwave ablation have been used to treat small PNETs. However, the use of these techniques is still controversial and requires further study.^102,103^

In the current study, we compared survival outcomes following surgery or following different types of nonsurgical management. Overall, we observed an almost 70% lower risk of mortality following surgical resection than following nonsurgical management. In agreement with these findings, Hill et al. reported a median survival of 114 months in patients with PNETs who underwent surgical resection compared with 35 months for those who were recommended for but did not undergo resection.^29^ These findings were backed up by those of another study, showing a median overall survival of 43 months for patients with PNETs who underwent surgical resection compared with 19 months for those who were treated nonsurgically.^104^

Surgery is considered necessary for PNET management for several reasons. First, surgical resection of the tumor is often the most effective way to remove it completely and minimize the risk of recurrence, which is common in PNETs.^105^ Complete surgical removal of the tumor can be curative, especially for early-stage tumors. Second, surgery can play a role in staging the tumor. Accurate staging is essential for determining the appropriate treatment approach and predicting the patient’s prognosis. Surgery allows the size and extent of the tumor to be measured accurately, as well as the assessment of lymph nodes and other structures around the tumor.^106^ Third, neuroendocrine tumors have a unique biology, which often requires individualized treatment. Surgery is often used in conjunction with other treatments, such as radiation therapy or chemotherapy, to achieve the best possible outcome for the patient.^107^ Surgery may also be necessary for metastatic PNETs, especially limited metastases that can be completely removed through surgery.^108^

However, nonsurgical approaches, such as systemic therapy or interventional radiology, can also be used to manage metastatic PNETs.^109^ We found that patients with metastatic PNETs who underwent surgical resection had a significantly lower risk of mortality than those who did not undergo surgery. In agreement with these findings, a retrospective single-center study showed that patients with nonfunctional metastatic PNETs receiving palliative or radical surgery had significantly better survival than patients who received nonsurgical treatment.^110^ However, the decision to use surgical or nonsurgical approaches for metastatic PNETs depends on various factors, including the size, location, and number of metastases, the overall health and medical history of the patient, and the patient’s treatment goals and preferences.

In this meta-analysis, we conducted subgroup analyses based on tumor size, tumor grade, and metastases. Small PNETs (< 2 cm) may be less likely to cause symptoms or spread to other parts of the body, and patients with small PNETs may not require immediate surgery. In these cases, observation and other nonsurgical approaches, such as active surveillance or periodic imaging, may be recommended instead.^111^ However, we found that patients with small PNETs who underwent surgical resection had better survival outcomes than those who did not undergo surgery. These findings are supported by those of Sharpe et al., who showed better survival in patients with localized small PNETs who underwent resection instead of observation.^41^ These findings suggest that surgery is a better option, even in cases of small and asymptomatic PNETs. In contrast to our findings for PNETs smaller than 2 cm, our analysis did not observe a significant survival benefit for surgical resection in patients with PNETs smaller than 1 cm. This suggests that nonsurgical management, such as active surveillance, may be a reasonable option for these smaller tumors. Therefore, these findings support a more conservative approach to the management of very small PNETs, especially when they are asymptomatic.

PNETs demonstrate distinct molecular subgroups with prognostic implications. One large study found that PNETs with ATRX/DAXX loss had significantly higher rates of nodal and distant metastases and markedly worse long-term survival than wild-type tumors. Five-year disease-free survival dropped from around 96% in ATRX/DAXX-intact cases to around 40% in ATRX/DAXX-deficient cases.^112^ Similarly, higher Ki-67 proliferation rate correlated with early recurrence.^113^ These molecular markers thus identify high-risk PNET subtypes warranting closer surveillance and adjuvant strategies. However, in our qualitative analysis, we found that molecular subgroup comparisons between surgical and nonsurgical management remain extremely limited. Notably, no study included in our review compared outcomes based on ATRX, DAXX, or telomere alterations between treatment groups. Further research is needed to clarify how molecular subtypes may influence the choice and effectiveness of treatment strategies. In addition, patients with hereditary syndromes can exhibit distinct PNET behavior and outcomes. For example, pancreatic NETs arising in VHL disease tend to be indolent: a cohort study noted superior long-term survival in patients with VHL after resection compared with matched sporadic cases.^114^ In contrast, MEN1 usually presents with multifocal PNETs and higher metastatic potential as tumors enlarge.^115^ Familial context thus informs a tailored approach, ranging from active surveillance of tiny lesions to timely resection in high-risk cases. However, our qualitative analysis revealed that only a few studies focused exclusively on patients with MEN1, with conflicting results regarding the benefit of surgery in small, nonfunctioning tumors. Moreover, most studies either did not report familial subgroups or lacked stratified outcome analyses, limiting firm conclusions on optimal treatment strategies in hereditary PNETs.

In regard to surgical management, most studies reported surgery as a binary variable without analyzing the outcomes of specific surgical techniques. Our qualitative analysis showed that less extensive resection can be appropriate for select PNETs. Enucleation of small, well-situated tumors in our series achieved oncologic outcomes comparable to formal resection, mirroring a multi-center study that found similar recurrence-free survival with enucleation versus standard pancreatectomy at the cost of a slightly higher pancreatic fistula rate.^116^ Notably, enucleation preserves pancreatic parenchyma and may reduce long-term endocrine insufficiency relative to distal pancreatectomy or the Whipple procedure. Thus, surgical approach (enucleation versus distal pancreatectomy versus pancreatoduodenectomy or even total pancreatectomy) should be individualized, balancing tumor factors against surgical morbidity to optimize outcomes. For nonsurgical management, strategies were rarely stratified by type, with most studies assumed them as a single category. Our review highlighted that somatostatin analogs and PRRT can achieve disease stabilization and symptom control in many cases. Chemotherapy remains vital for progressive disease. A recent randomized trial in metastatic PNETs demonstrated significantly prolonged median progression-free survival with capecitabine-temozolomide combination (22.7 months) versus temozolomide alone (14.4 months).^117^ This underscores the efficacy of modern medical regimens. In addition, in select patients with small indolent tumors, careful observation is a viable initial strategy. The optimal approach is thus personalized on the basis of tumor burden and growth rate.

In the concept of tumor location, most studies did not stratify outcomes by tumor location, but body/tail PNETs were more often resected and associated with better overall survival, while head tumors were linked to higher surgical risk and poorer conservative outcomes. Only a few studies demonstrated a clear survival advantage of surgery for specific tumor locations, particularly in the tail. Lesions in the pancreatic head often present at larger size and with nodal involvement, potentially portending a worse prognosis than those in the body/tail. A population-based analysis found that 2–4 cm PNETs in the head had significantly lower survival than size-matched tumors in the body/tail.^118^ These disparities likely reflect both tumor biology and the greater complexity of resecting head tumors (Whipple procedures) versus distal pancreatectomy. Moreover, in the assessment of cause-specific mortality, direct comparisons between surgical and nonsurgical groups are limited, but tumor progression and liver metastases were the leading causes of death in both. Patients who underwent resection had low rates of tumor-related mortality (often surviving long enough to die of other causes), whereas those managed nonsurgically more often succumbed to disease progression (e.g., liver metastatic burden).

Taken together, evidence suggests that surgical management is the better choice for managing patients with PNETs. The European Society of Endocrine Surgeons (ESES) recommends surgery for advanced PNETs, as discussed at a consensus meeting.^13^ In addition, the North American Neuroendocrine Tumor Society (NANETS), the European Neuroendocrine Tumor Society (ENETS), and the American Association of Endocrine Surgeons (AAES) all recommend surgical resection for functional tumors and nonfunctional tumors > 2 cm, with observation reserved for select small, asymptomatic cases.^119,120^ The agreement between our data-driven findings and expert consensus highlights the importance of evidence-based expert opinion in guiding clinical decision-making. This is particularly valuable in complex cases such as PNETs, where empirical evidence alone may not fully capture the complexity of patient management.

Our study had certain limitations. First, there was heterogeneity in our results. To address this, we used the GRADE tool and acknowledged that the retrieved results presented a low level of evidence. Second, all included studies were observational and retrospective in nature. To improve the quality of evidence, randomized clinical trials or matched case-control studies are needed to compare the efficacy of surgical and nonsurgical management. Furthermore, current evidence does not consistently report the functional status of tumors (e.g., insulinoma, gastrinoma, glucagonoma), and most studies did not stratify outcomes by specific functional subtypes. It is also important to note that functional PNETs are typically treated surgically, even at small sizes, which likely leads to underrepresentation of these tumors in the nonsurgical comparator group. As a result, survival outcomes for nonsurgically managed functional PNETs may be underestimated. Nevertheless, we performed a subgroup analysis only in functional PNETs, and included and analyzed them accordingly in our quantitative synthesis. This limitation highlights the need for more granular, function-specific comparative data in future research. Another limitation is that several included studies enrolled patients diagnosed prior to the widespread adoption of modern World Health Organization (WHO) grading and classification systems. Since most studies did not stratify outcomes by diagnostic era or histologic criteria, older cases with potentially inconsistent diagnostic standards may have been included. However, many of these studies were excluded through sensitivity analyses based on sample size and follow-up duration, minimizing the impact of historical heterogeneity. Future studies should stratify outcomes by diagnostic standards to improve comparability. The final limitation is that for many key outcomes, including molecular subtypes, hereditary syndromes, optimal surgical/nonsurgical managements, tumor location, and cause-specific mortality, subgroup stratification was not performed in the majority of included studies, making formal meta-analysis infeasible. To address this gap, we conducted a qualitative synthesis of studies that did report these subgroup data in order to provide a narrative evaluation of these important clinical variables. These limitations highlight the need for future studies to systematically stratify and report such subgroups to enable more granular and informative comparative analyses.

## Conclusions

Surgical management of functional, nonfunctional, low and moderate grade, small, and metastatic/locally advanced PNETs is associated with better survival outcomes than nonsurgical management is. However, for < 1 cm, asymptomatic PNETs, nonsurgical management may be considered a reasonable alternative, given the comparable long-term survival rates. These quantitative findings support some aspects of qualitative opinions in the field. Further prospective evidence is needed to validate these findings and explore the potential benefits of managing PNETs surgically.

## Supplementary Information

Below is the link to the electronic supplementary material.

Supplementary file1 (DOCX 420 KB)

## Data Availability

Data used in this study is available upon request to the corresponding author.
