# Supplementary material for: Surgical Versus Nonsurgical Management of Pancreatic Neuroendocrine Tumors: A Systematic Review and Meta-Analysis
Source: Ann Surg Oncol. 2025 Jul 24;32(10):7532–49. doi: 10.1245/s10434-025-17819-3 (PMC12454575; doi:10.1245/s10434-025-17819-3)
Supplement: Supplementary file 1 — Supplementary file1 (DOCX 420 KB) [file 10434_2025_17819_MOESM1_ESM.docx]

**Supplementary Materials**

**Surgical versus non-surgical management of pancreatic neuroendocrine tumors: a systematic review and meta-analysis**

Elias Khajeh^1^, MD, MPH; Mohammadamin Shahrbaf^1^, MD; Leonidas Apostolidis^2^, MD;

Christoph W Michalski^1^, MD; Martin Loos^1^, MD; Arianeb Mehrabi^1*^, MD

^1^ Department of General, Visceral and Transplantation Surgery, Heidelberg University, Heidelberg, Germany.

^2^ National Center for Tumor Diseases (NCT) Heidelberg, Department of Medical Oncology, Heidelberg University Hospital, Heidelberg, Germany.

***Correspondence to:** Professor Arianeb Mehrabi, MD, FICS, FACS, FEBS

Head of the Division of Liver Surgery, Department of General, Visceral, and Transplantation Surgery, University of Heidelberg, Im Neuenheimer Feld 420, 69120 Heidelberg, Germany

**Tel:** 0049 – 6221 - 5636223

**Fax:** 0049 – 6221 - 565781

**E-Mail:** [arianeb.mehrabi@med.uni-heidelberg.de](mailto:arianeb.mehrabi@med.uni-heidelberg.de)

**Headline Page number**

Supplementary method 3

Supplementary results 9

**Supplementary method**

**Search Strategy**

The search was performed by combining three groups of search terms: Group A included terms related to surgical interventions such as "surgical", "surgery", "operation", "resection", "enucleation", "pancreatectomy", and "pancreatoduodenectomy". Group B included terms related to non-surgical interventions such as "conservative", "wait and watch", "chemotherapy", "endoscopy", "EUS", "interventional radiology", "drug therapy", "radiotherapy", "molecular targeted therapy", "ablation", "radiofrequency", "embolization", and "chemoembolization". Group C included terms related to pancreatic neuroendocrine tumors such as "neuroendocrine tumor", "gastroenteropancreatic", "PNET", "P-NET", "panNET", and "pan-NET". The search was conducted using "AND" to combine the keywords within the Title/Abstract or Title/Abstract/Keywords fields, and the final search was performed on 31 December 2023. Search query in each database is provided below:

**PubMed:** (("surgical"[Title/Abstract] OR "surgery"[Title/Abstract] OR "operation"[Title/Abstract] OR "resection"[Title/Abstract] OR "enucleation"[Title/Abstract] OR "pancreatectomy"[Title/Abstract] OR "pancreatoduodenectomy"[Title/Abstract]) AND ("non-surgical"[Title/Abstract] OR "nonsurgical"[Title/Abstract] OR "conservative"[Title/Abstract] OR "wait and watch"[Title/Abstract] OR "non surgery"[Title/Abstract] OR "chemotherapy"[Title/Abstract] OR "endoscopy"[Title/Abstract] OR "EUS"[Title/Abstract] OR "interventional radiology"[Title/Abstract] OR "drug therapy"[Title/Abstract] OR "radiotherapy"[Title/Abstract] OR "molecular targeted therapy"[Title/Abstract] OR "ablation"[Title/Abstract] OR "radiofrequency"[Title/Abstract] OR "embolization"[Title/Abstract] OR "chemoembolization"[Title/Abstract])) AND ("pancreas neuroendocrine tumor"[Title/Abstract] OR "neuroendocrine tumor"[Title/Abstract] OR "gastroenteropancreatic"[Title/Abstract] OR "PNET"[Title/Abstract] OR "P-NET"[Title/Abstract] OR "panNET"[Title/Abstract] OR "pan-NET"[Title/Abstract])

**Scopus:** ( TITLE-ABS-KEY ( "surgical" OR "surgery" OR "operation" OR "resection" OR "enucleation" OR "pancreatectomy" OR "pancreatoduodenectomy" ) AND TITLE-ABS-KEY ( "non-surgical" OR "nonsurgical" OR "conservative" OR "wait and watch" OR "non surgery" OR "chemotherapy" OR "endoscopy" OR "EUS" OR "interventional radiology" OR "drug therapy" OR "radiotherapy" OR "molecular targeted therapy" OR "ablation" OR "radiofrequency" OR "embolization" OR "chemoembolization" ) AND TITLE-ABS-KEY ( "pancreas neuroendocrine tumor" OR "neuroendocrine tumor" OR "gastroenteropancreatic" OR "PNET" OR "P-NET" OR "panNET" OR "pan-NET" ) )

**Web of Science:** “surgical” OR “surgery” OR “operation” OR “resection” OR “enucleation” OR “pancreatectomy” OR “pancreatoduodenectomy” (Topic) AND “non-surgical” OR “nonsurgical” OR “conservative” OR “wait and watch” OR “non surgery” OR “chemotherapy” OR “endoscopy” OR “EUS” OR “interventional radiology” OR “drug therapy” OR “radiotherapy” OR “molecular targeted therapy” OR “ablation” OR “radiofrequency” OR “embolization” OR “chemoembolization” (Topic) AND “pancreas neuroendocrine tumor” OR “neuroendocrine tumor” OR “gastroenteropancreatic” OR “PNET” OR “P-NET” OR “panNET” OR “pan-NET” (Topic)

**Embase:** ('surgical':ti,ab,kw OR 'surgery':ti,ab,kw OR 'operation':ti,ab,kw OR 'resection':ti,ab,kw OR 'enucleation':ti,ab,kw OR 'pancreatectomy':ti,ab,kw OR 'pancreatoduodenectomy':ti,ab,kw) AND (('non-surgical':ti,ab,kw OR 'nonsurgical':ti,ab,kw OR 'conservative':ti,ab,kw OR wait:ti,ab,kw) AND watch:ti,ab,kw OR 'non surgery':ti,ab,kw OR 'chemotherapy':ti,ab,kw OR 'endoscopy':ti,ab,kw OR 'eus':ti,ab,kw OR 'interventional radiology':ti,ab,kw OR 'drug therapy':ti,ab,kw OR 'radiotherapy':ti,ab,kw OR 'molecular targeted therapy':ti,ab,kw OR 'ablation':ti,ab,kw OR 'radiofrequency':ti,ab,kw OR 'embolization':ti,ab,kw OR 'chemoembolization':ti,ab,kw) AND ('pancreas neuroendocrine tumor':ti,ab,kw OR 'neuroendocrine tumor':ti,ab,kw OR 'gastroenteropancreatic':ti,ab,kw OR 'pnet':ti,ab,kw OR 'p-net':ti,ab,kw OR 'pannet':ti,ab,kw OR 'pan-net':ti,ab,kw)

PRISMA 2020 checklist

| **Section and Topic** | **Item #** | **Checklist item** | **Location where item is reported** |
| --- | --- | --- | --- |
| **TITLE** | | |  |
| Title | 1 | Identify the report as a systematic review. | Page 1 |
| **ABSTRACT** | | |  |
| Abstract | 2 | See the PRISMA 2020 for Abstracts checklist. | Page 4,5 |
| **INTRODUCTION** | | |  |
| Rationale | 3 | Describe the rationale for the review in the context of existing knowledge. | Page 6 |
| Objectives | 4 | Provide an explicit statement of the objective(s) or question(s) the review addresses. | Page 7 |
| **METHODS** | | |  |
| Eligibility criteria | 5 | Specify the inclusion and exclusion criteria for the review and how studies were grouped for the syntheses. | Page 9 |
| Information sources | 6 | Specify all databases, registers, websites, organisations, reference lists and other sources searched or consulted to identify studies. Specify the date when each source was last searched or consulted. | Page 8 |
| Search strategy | 7 | Present the full search strategies for all databases, registers and websites, including any filters and limits used. | Page 8,9 |
| Selection process | 8 | Specify the methods used to decide whether a study met the inclusion criteria of the review, including how many reviewers screened each record and each report retrieved, whether they worked independently, and if applicable, details of automation tools used in the process. | Page 9,10 |
| Data collection process | 9 | Specify the methods used to collect data from reports, including how many reviewers collected data from each report, whether they worked independently, any processes for obtaining or confirming data from study investigators, and if applicable, details of automation tools used in the process. | Page 9,10 |
| Data items | 10a | List and define all outcomes for which data were sought. Specify whether all results that were compatible with each outcome domain in each study were sought (e.g. for all measures, time points, analyses), and if not, the methods used to decide which results to collect. | Page 9 |
|  | 10b | List and define all other variables for which data were sought (e.g. participant and intervention characteristics, funding sources). Describe any assumptions made about any missing or unclear information. | Page 9 |
| Study risk of bias assessment | 11 | Specify the methods used to assess risk of bias in the included studies, including details of the tool(s) used, how many reviewers assessed each study and whether they worked independently, and if applicable, details of automation tools used in the process. | Page 10 |
| Effect measures | 12 | Specify for each outcome the effect measure(s) (e.g. risk ratio, mean difference) used in the synthesis or presentation of results. | Page 10 |
| Synthesis methods | 13a | Describe the processes used to decide which studies were eligible for each synthesis (e.g. tabulating the study intervention characteristics and comparing against the planned groups for each synthesis (item #5)). | Page 9,10 |
|  | 13b | Describe any methods required to prepare the data for presentation or synthesis, such as handling of missing summary statistics, or data conversions. | Page 9,10 |
|  | 13c | Describe any methods used to tabulate or visually display results of individual studies and syntheses. | Page 9,10 |
|  | 13d | Describe any methods used to synthesize results and provide a rationale for the choice(s). If meta-analysis was performed, describe the model(s), method(s) to identify the presence and extent of statistical heterogeneity, and software package(s) used. | Page 9,10 |
|  | 13e | Describe any methods used to explore possible causes of heterogeneity among study results (e.g. subgroup analysis, meta-regression). | Page 9,10 |
|  | 13f | Describe any sensitivity analyses conducted to assess robustness of the synthesized results. | Page 9,10 |
| Reporting bias assessment | 14 | Describe any methods used to assess risk of bias due to missing results in a synthesis (arising from reporting biases). | Page 9,10 |
| Certainty assessment | 15 | Describe any methods used to assess certainty (or confidence) in the body of evidence for an outcome. | Page 9,10 |
| **RESULTS** | | |  |
| Study selection | 16a | Describe the results of the search and selection process, from the number of records identified in the search to the number of studies included in the review, ideally using a flow diagram. | Page 11 |
|  | 16b | Cite studies that might appear to meet the inclusion criteria, but which were excluded, and explain why they were excluded. | Page 11 |
| Study characteristics | 17 | Cite each included study and present its characteristics. | Page 11, Table 1 |
| Risk of bias in studies | 18 | Present assessments of risk of bias for each included study. | Page 11, Table S1 |
| Results of individual studies | 19 | For all outcomes, present, for each study: (a) summary statistics for each group (where appropriate) and (b) an effect estimate and its precision (e.g. confidence/credible interval), ideally using structured tables or plots. | Page 11-21 |
| Results of syntheses | 20a | For each synthesis, briefly summarise the characteristics and risk of bias among contributing studies. | Page 11-21 |
|  | 20b | Present results of all statistical syntheses conducted. If meta-analysis was done, present for each the summary estimate and its precision (e.g. confidence/credible interval) and measures of statistical heterogeneity. If comparing groups, describe the direction of the effect. | Page 11-21 |
|  | 20c | Present results of all investigations of possible causes of heterogeneity among study results. | Page 11-21 |
|  | 20d | Present results of all sensitivity analyses conducted to assess the robustness of the synthesized results. | Page 11-21 |
| Reporting biases | 21 | Present assessments of risk of bias due to missing results (arising from reporting biases) for each synthesis assessed. | Page 22 |
| Certainty of evidence | 22 | Present assessments of certainty (or confidence) in the body of evidence for each outcome assessed. | Page 22 |
| **DISCUSSION** | | |  |
| Discussion | 23a | Provide a general interpretation of the results in the context of other evidence. | Page 23 |
|  | 23b | Discuss any limitations of the evidence included in the review. | Page 28,29 |
|  | 23c | Discuss any limitations of the review processes used. | Page 28,29 |
|  | 23d | Discuss implications of the results for practice, policy, and future research. | Page 28,29 |
| **OTHER INFORMATION** | | |  |
| Registration and protocol | 24a | Provide registration information for the review, including register name and registration number, or state that the review was not registered. | Page 8 |
|  | 24b | Indicate where the review protocol can be accessed, or state that a protocol was not prepared. | Page 8 |
|  | 24c | Describe and explain any amendments to information provided at registration or in the protocol. | Page 8 |
| Support | 25 | Describe sources of financial or non-financial support for the review, and the role of the funders or sponsors in the review. | Page 2 |
| Competing interests | 26 | Declare any competing interests of review authors. | Page 2 |
| Availability of data, code and other materials | 27 | Report which of the following are publicly available and where they can be found: template data collection forms; data extracted from included studies; data used for all analyses; analytic code; any other materials used in the review. | Page 2 |

*From:*  Page MJ, McKenzie JE, Bossuyt PM, Boutron I, Hoffmann TC, Mulrow CD, et al. The PRISMA 2020 statement: an updated guideline for reporting systematic reviews. BMJ 2021;372:n71. doi: 10.1136/bmj.n71. This work is licensed under CC BY 4.0. To view a copy of this license, visit <https://creativecommons.org/licenses/by/4.0/>

**Supplementary results**

**Table S1.** Risk of bias assessment

| Study | Confounding | Selection of Participants | Classification of Interventions | Deviations from Intended Interventions | Missing Data | Measurement of Outcomes | Selective Reporting | Overall |
| --- | --- | --- | --- | --- | --- | --- | --- | --- |
| Solorzano et al. 2001 | Serious | Serious | Low | Moderate | Serious | Low | Low | Serious |
| Chu et al. 2002 | Serious | Serious | Low | Moderate | Low | Low | Low | Serious |
| Tomassetti et al. 2005 | Serious | Serious | Low | Moderate | Low | Low | Low | Serious |
| Kouvaraki et al. 2006 | Serious | Serious | Low | Moderate | Low | Low | Low | Serious |
| Triponez et al. 2006 | Serious | Serious | Low | Moderate | Serious | Low | Moderate | Serious |
| Nguyen et al. 2007 | Serious | Serious | Low | Moderate | Low | Low | Moderate | Serious |
| Shurr et al. 2007 | Serious | Serious | Moderate | Moderate | Low | Low | Low | Serious |
| Fischer et al. 2008 | Serious | Serious | Low | Moderate | Serious | Low | Low | Serious |
| Ruiz-Tovar et al. 2008 | Serious | Serious | Moderate | Moderate | Moderate | Low | Moderate | Serious |
| Bettini et al. 2009 | Moderate | Serious | Low | Low | Moderate | Low | Moderate | Serious |
| Hill et al. 2009 | Serious | Serious | Low | Moderate | Low | Low | Low | Serious |
| Franko et al. 2010 | Serious | Serious | Moderate | Moderate | Serious | Low | Low | Serious |
| Ito et al. 2010 | Serious | Serious | Low | Moderate | Low | Low | Moderate | Serious |
| Wang et al. 2011 | Serious | Serious | Moderate | Moderate | Low | Low | Moderate | Serious |
| Martin-Perez et al. 2013 | Serious | Serious | Moderate | Moderate | Low | Low | Low | Serious |
| Zerbi et al. 2013 | Serious | Serious | Low | Moderate | Serious | Low | Low | Serious |
| Bertani et al. 2014 | Moderate | Serious | Low | Moderate | Moderate | Moderate | Moderate | Serious |
| Crippa et al. 2014 | Serious | Serious | Low | Moderate | Serious | Low | Moderate | Serious |
| Gratian et al. 2014 | Serious | Serious | Low | Moderate | Low | Low | Low | Serious |
| Dumont et al. 2015 | Moderate | Serious | Low | Moderate | Low | Low | Moderate | Serious |
| Hüttner et al. 2015 | Serious | Serious | Low | Moderate | Low | Low | Low | Serious |
| Partelli et al. 2015 | Serious | Serious | Low | Moderate | Low | Low | Low | Serious |
| Sharpe et al. 2015 | Serious | Serious | Low | Moderate | Low | Low | Low | Serious |
| van Vliet et al. 2015 | Moderate | Serious | Low | Moderate | Low | Low | Low | Serious |
| Bertani et al. 2016 | Moderate | Serious | Low | Moderate | Low | Low | Moderate | Serious |
| Haugvik et al. 2016 | Serious | Serious | Moderate | Moderate | Serious | Low | Low | Serious |
| Keutgen et al.a 2016 | Serious | Serious | Moderate | Moderate | Low | Low | Low | Serious |
| Keutgen et al.b 2016 | Moderate | Serious | Moderate | Moderate | Serious | Low | Low | Serious |
| Massironi et al. 2016 | Serious | Serious | Moderate | Moderate | Moderate | Low | Moderate | Serious |
| Partelli et al. 2016 | Serious | Serious | Moderate | Moderate | Low | Low | Moderate | Serious |
| Rosenberg et al. 2016 | Low | Serious | Low | Moderate | Low | Low | Moderate | Serious |
| Sadot et al. 2016 | Low | Serious | Moderate | Moderate | Low | Low | Moderate | Serious |
| Zhang et al. 2016 | Serious | Serious | Moderate | Moderate | Low | Low | Low | Serious |
| Bertani et al. 2017 | Moderate | Low | Low | Moderate | Low | Low | Low | Moderate |
| Citterio et al. 2017 | Moderate | Serious | Moderate | Moderate | Serious | Low | Low | Serious |
| Prakash et al. 2017 | Moderate | Serious | Low | Moderate | Low | Low | Low | Serious |
| Tao et al. 2017 | Serious | Serious | Moderate | Moderate | Low | Low | Low | Serious |
| Chawla et al. 2018 | Moderate | Serious | Moderate | Low | Low | Low | Low | Serious |
| Genc et al. 2018 | Moderate | Moderate | Low | Low | Moderate | Low | Low | Moderate |
| Nell et al. 2018 | Serious | Moderate | Moderate | Moderate | Low | Low | Low | Serious |
| Li et al. 2019 | Serious | Serious | Moderate | Moderate | Low | Low | Low | Serious |
| Tierney et al. 2019 | Low | Serious | Moderate | Moderate | Low | Low | Low | Serious |
| Wu et al. 2019 | Moderate | Serious | Moderate | Moderate | Serious | Low | Low | Serious |
| Ye et al. 2019 | Low | Serious | Low | Moderate | Moderate | Low | Low | Serious |
| Zhang et al. 2019 | Moderate | Serious | Low | Moderate | Moderate | Low | Low | Serious |
| Zheng et al. 2019 | Low | Serious | Low | Moderate | Moderate | Low | Low | Serious |
| Assi et al. 2020 | Serious | Serious | Moderate | Moderate | Moderate | Low | Low | Serious |
| Chivukula et al. 2020 | Moderate | Serious | Moderate | Moderate | Moderate | Low | Low | Serious |
| Fathi et al. 2020 | Serious | Serious | Moderate | Moderate | Moderate | Low | Low | Serious |
| Fujimori et al. 2020 | Low | Serious | Moderate | Moderate | Moderate | Low | Low | Serious |
| Kurita et al. 2020 | Moderate | Serious | Moderate | Moderate | Moderate | Moderate | Low | Serious |
| Powers et al. 2020 | Moderate | Serious | Moderate | Moderate | Low | Low | Low | Serious |
| Sada et al. 2020 | Low | Serious | Moderate | Moderate | Low | Low | Low | Serious |
| Kaemmerer et al. 2021 | Moderate | Serious | Moderate | Moderate | Moderate | Moderate | Low | Serious |
| Shaib et al. 2021 | Low | Serious | Moderate | Moderate | Low | Low | Low | Serious |
| Sun et al. 2021 | Serious | Serious | Moderate | Moderate | Low | Low | Low | Serious |
| Tsilimigars et al. 2021 | Serious | Serious | Moderate | Moderate | Moderate | Low | Low | Serious |
| Bingmer et al. 2022 | Serious | Serious | Moderate | Moderate | Low | Low | Low | Serious |
| Cai et al. 2022 | Serious | Serious | Moderate | Moderate | Moderate | Low | Low | Serious |
| Guo et al. 2022 | Serious | Serious | Moderate | Moderate | Moderate | Low | Low | Serious |
| Kaur et al. 2022 | Serious | Serious | Low | Moderate | Moderate | Low | Low | Serious |
| Kjaer et al. 2022 | Moderate | Serious | Low | Moderate | Moderate | Low | Moderate | Serious |
| Krogh et al. 2022 | Moderate | Serious | Low | Moderate | Low | Low | Low | Serious |
| Liu et al. 2022 | Serious | Serious | Moderate | Moderate | Moderate | Low | Moderate | Serious |
| Minczeles et al. 2022 | Moderate | Serious | Moderate | Moderate | Low | Low | Moderate | Serious |
| Mou et al. 2022 | Moderate | Serious | Moderate | Moderate | Low | Low | Low | Serious |
| So et al. 2022 | Low | Low | Low | Low | Low | Low | Low | Low |
| Wu et al. 2022 | Moderate | Serious | Moderate | Moderate | Moderate | Low | Low | Serious |
| Yang et al.a 2022 | Low | Serious | Moderate | Moderate | Moderate | Low | Low | Serious |
| Zhu et al. 2022 | Low | Serious | Low | Moderate | Moderate | Low | Low | Serious |
| Yang et al.b 2022 | Serious | Serious | Moderate | Moderate | Low | Low | Low | Serious |
| Ye et al. 2022 | Low | Serious | Low | Low | Low | Low | Low | Serious |
| Amini et al. 2023 | Serious | Serious | Moderate | Moderate | Low | Low | Low | Serious |
| Crinò et al. 2023 | Low | Serious | Low | Low | Low | Low | Moderate | Serious |
| Heng et al. 2023 | Serious | Serious | Moderate | Moderate | Low | Moderate | Moderate | Serious |
| Lin et al. 2023 | Low | Serious | Low | Low | Low | Low | Low | Serious |
| Luo et al. 2023 | Moderate | Serious | Moderate | Moderate | Low | Low | Moderate | Serious |

**
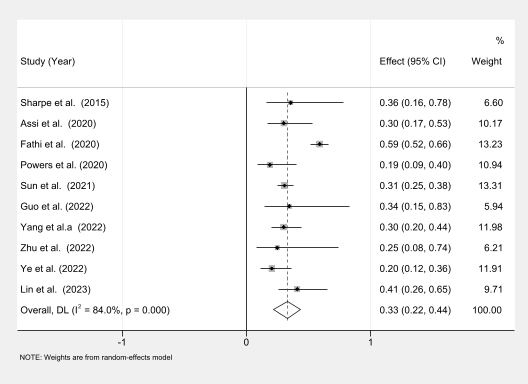
Figure S1.** Mortality hazard ratio for surgical management in nonfunctional < 2cm PNETs

**Subgroup analysis of G1 PNETs**

A pooled analysis of six studies comprising 3 879 patients was conducted to determine the mortality HR of surgical versus non-surgical management for G1 (well-differentiated) PNETs. The HR was 0.27 (95% CI, 0.11–0.43; P = 0.001). After analyzing six studies with a total of 3 353 patients, the 1-year survival rate was 94% (95% CI, 90–98%) for surgical management and 78% (95% CI, 73–82%) for non-surgical management. Surgical management was associated with a 3-year survival rate of 86% (95% CI, 75–97%) while non-surgical management was associated with a 3-year survival rate of 51% (95% CI, 43–59%). Surgical management was associated with a 5-year survival rate of 76% (95% CI, 65–87%) while non-surgical management was associated with a 5-year survival rate of 39% (95% CI, 31–46%). Of the studies that reported 10-year survival, surgical management was associated with a 10-year survival rate of 78% (95% CI, 56–100%) while non-surgical management was associated with a 10-year survival rate of 26% (95% CI, 2–50%).


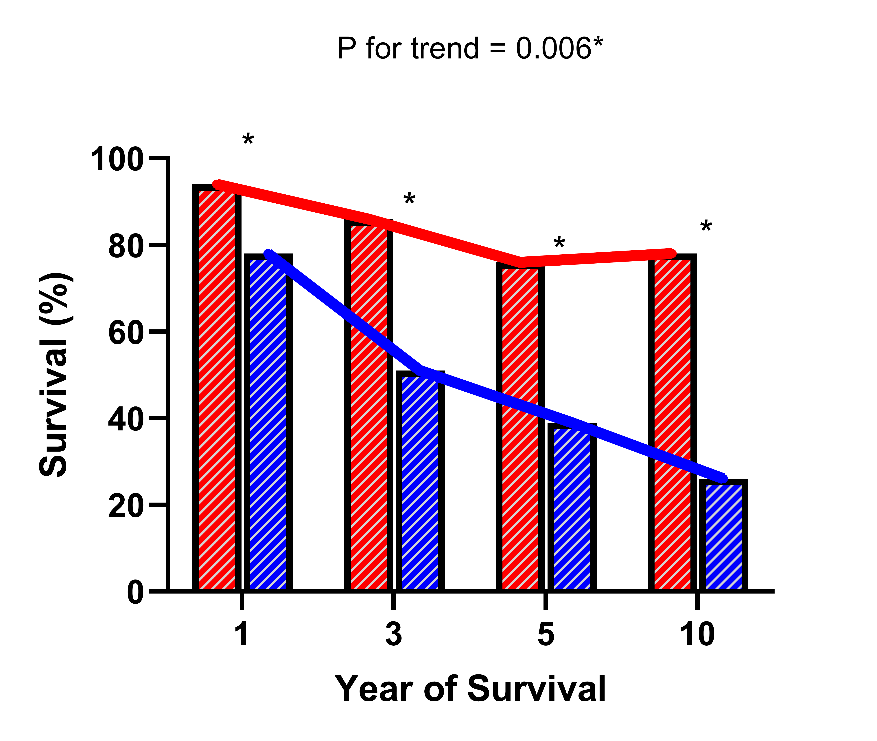

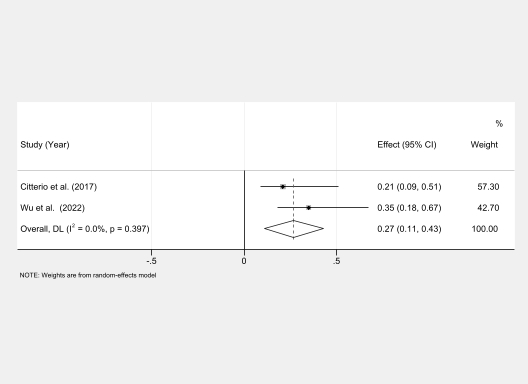


**Figure S2.** Mortality hazard ratio and survival rate and trend for G1 PNETs


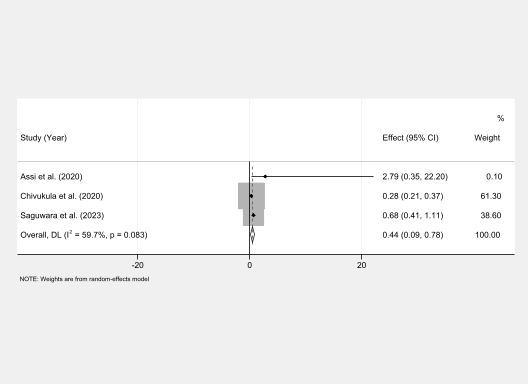
**Figure S3.** Mortality hazard ratio for surgical management in < 1cm PNETs

**Table S2. GRADE chart for quality of evidence**

| **Outcome** | **Risk of bias** | **Inconsistency** | **Indirectness** | **Imprecision** | **Publication bias** | **Quality** |
| --- | --- | --- | --- | --- | --- | --- |
| HR for surgery (overall) | Serious limitation | Very Serious limitation | Serious limitation | No serious limitation | Serious limitation | Very low |
| 1-year survival surgery (overall) | Serious limitation | Very Serious limitation | Serious limitation | No serious limitation | Serious limitation | Very low |
| 1-year survival non-surgery (overall) | Serious limitation | Very Serious limitation | Serious limitation | No serious limitation | No serious limitation | Low |
| 3-year survival surgery (overall) | Serious limitation | Very Serious limitation | Serious limitation | No serious limitation | Serious limitation | Very low |
| 3-year survival non-surgery (overall) | Serious limitation | Very Serious limitation | Serious limitation | No serious limitation | No serious limitation | Low |
| 5-year survival surgery (overall) | Serious limitation | Very Serious limitation | Serious limitation | No serious limitation | Serious limitation | Very low |
| 5-year survival non-surgery (overall) | Serious limitation | Very Serious limitation | Serious limitation | No serious limitation | No serious limitation | Low |
| 10-year survival surgery (overall) | Serious limitation | Very Serious limitation | Serious limitation | No serious limitation | No serious limitation | Low |
| 10-year survival non-surgery (overall) | Serious limitation | Very Serious limitation | Serious limitation | No serious limitation | No serious limitation | Low |
| HR for surgery (functional) | Serious limitation | Serious limitation | Serious limitation | No serious limitation | No serious limitation | Low |
| 1-year survival surgery (functional) | Serious limitation | Very Serious limitation | Serious limitation | No serious limitation | No serious limitation | Low |
| 1-year survival non-surgery (functional) | Serious limitation | Very Serious limitation | Serious limitation | No serious limitation | No serious limitation | Low |
| 3-year survival surgery (functional) | Serious limitation | Very Serious limitation | Serious limitation | No serious limitation | No serious limitation | Low |
| 3-year survival non-surgery (functional) | Serious limitation | Very Serious limitation | Serious limitation | No serious limitation | No serious limitation | Low |
| 5-year survival surgery (functional) | Serious limitation | Very Serious limitation | Serious limitation | No serious limitation | No serious limitation | Low |
| 5-year survival non-surgery (functional) | Serious limitation | Very Serious limitation | Serious limitation | No serious limitation | No serious limitation | Low |
| 10-year survival surgery (functional) | Serious limitation | Very Serious limitation | Serious limitation | No serious limitation | No serious limitation | Low |
| 10-year survival non-surgery (functional) | Serious limitation | Very Serious limitation | Serious limitation | No serious limitation | No serious limitation | Low |
| HR for surgery (non-functional) | Serious limitation | Very Serious limitation | Serious limitation | No serious limitation | No serious limitation | Low |
| 1-year survival surgery (non-functional) | Serious limitation | Very Serious limitation | Serious limitation | No serious limitation | No serious limitation | Low |
| 1-year survival non-surgery (non-functional) | Serious limitation | Very Serious limitation | Serious limitation | No serious limitation | No serious limitation | Low |
| 3-year survival surgery (non-functional) | Serious limitation | Very Serious limitation | Serious limitation | No serious limitation | No serious limitation | Low |
| 3-year survival non-surgery (non-functional) | Serious limitation | Very Serious limitation | Serious limitation | No serious limitation | No serious limitation | Low |
| 5-year survival surgery (non-functional) | Serious limitation | Very Serious limitation | Serious limitation | No serious limitation | No serious limitation | Low |
| 5-year survival non-surgery (non-functional) | Serious limitation | Very Serious limitation | Serious limitation | No serious limitation | No serious limitation | Low |
| 10-year survival surgery (non-functional) | Serious limitation | Very Serious limitation | Serious limitation | No serious limitation | No serious limitation | Low |
| 10-year survival non-surgery (non-functional) | Serious limitation | Very Serious limitation | Serious limitation | No serious limitation | No serious limitation | Low |
| HR for surgery (G1/G2) | Serious limitation | No serious limitation | Serious limitation | No serious limitation | No serious limitation | Low |
| 1-year survival surgery (G1/G2) | Serious limitation | Very Serious limitation | Serious limitation | No serious limitation | No serious limitation | Low |
| 1-year survival non-surgery (G1/G2) | Serious limitation | Very Serious limitation | Serious limitation | No serious limitation | No serious limitation | Low |
| 3-year survival surgery (G1/G2) | Serious limitation | Very Serious limitation | Serious limitation | No serious limitation | No serious limitation | Low |
| 3-year survival non-surgery (G1/G2) | Serious limitation | Very Serious limitation | Serious limitation | No serious limitation | No serious limitation | Low |
| 5-year survival surgery (G1/G2) | Serious limitation | Very Serious limitation | Serious limitation | No serious limitation | No serious limitation | Low |
| 5-year survival non-surgery (G1/G2) | Serious limitation | Very Serious limitation | Serious limitation | No serious limitation | No serious limitation | Low |
| 10-year survival surgery (G1/G2) | Serious limitation | Very Serious limitation | Serious limitation | No serious limitation | No serious limitation | Low |
| 10-year survival non-surgery (G1/G2) | Serious limitation | Very Serious limitation | Serious limitation | No serious limitation | No serious limitation | Low |
| HR for surgery (<2cm) | Serious limitation | No serious limitation | Serious limitation | No serious limitation | No serious limitation | Low |
| 1-year survival surgery (<2cm) | Serious limitation | Very Serious limitation | Serious limitation | No serious limitation | No serious limitation | Low |
| 1-year survival non-surgery (<2cm) | Serious limitation | Very Serious limitation | Serious limitation | No serious limitation | No serious limitation | Low |
| 3-year survival surgery (<2cm) | Serious limitation | Very Serious limitation | Serious limitation | No serious limitation | No serious limitation | Low |
| 3-year survival non-surgery (<2cm) | Serious limitation | Very Serious limitation | Serious limitation | No serious limitation | No serious limitation | Low |
| 5-year survival surgery (<2cm) | Serious limitation | Very Serious limitation | Serious limitation | No serious limitation | No serious limitation | Low |
| 5-year survival non-surgery (<2cm) | Serious limitation | Very Serious limitation | Serious limitation | No serious limitation | No serious limitation | Low |
| 10-year survival surgery (<2cm) | Serious limitation | Very Serious limitation | Serious limitation | No serious limitation | No serious limitation | Low |
| 10-year survival non-surgery (<2cm) | Serious limitation | Very Serious limitation | Serious limitation | No serious limitation | No serious limitation | Low |
| HR for surgery (metastatic) | Serious limitation | No serious limitation | Serious limitation | No serious limitation | No serious limitation | Low |
| 1-year survival surgery (metastatic) | Serious limitation | Very Serious limitation | Serious limitation | No serious limitation | No serious limitation | Low |
| 1-year survival non-surgery (metastatic) | Serious limitation | Very Serious limitation | Serious limitation | No serious limitation | No serious limitation | Low |
| 3-year survival surgery (metastatic) | Serious limitation | Very Serious limitation | Serious limitation | No serious limitation | No serious limitation | Low |
| 3-year survival non-surgery (metastatic) | Serious limitation | Very Serious limitation | Serious limitation | No serious limitation | No serious limitation | Low |
| 5-year survival surgery (metastatic) | Serious limitation | Very Serious limitation | Serious limitation | No serious limitation | No serious limitation | Low |
| 5-year survival non-surgery (metastatic) | Serious limitation | Very Serious limitation | Serious limitation | No serious limitation | No serious limitation | Low |
| 10-year survival surgery (metastatic) | Serious limitation | Very Serious limitation | Serious limitation | No serious limitation | No serious limitation | Low |
| 10-year survival non-surgery (metastatic) | Serious limitation | Very Serious limitation | Serious limitation | No serious limitation | No serious limitation | Low |
